# Motivational profiles regarding biology amongst German upper secondary students and associations with perceived basic need satisfaction and pressure

**DOI:** 10.3389/fpsyg.2026.1588742

**Published:** 2026-03-04

**Authors:** Lisa-Maria Kamps, Axel Grund, Matthias Wilde, Nadine Großmann

**Affiliations:** 1 Faculty of Biology, Biology Didactics, Bielefeld University, Bielefeld, Germany; 2 Department of Human Sciences, University of Luxembourg, Esch-sur-Alzette, Luxembourg; 3 Institute for Biology Education, University of Cologne, Cologne, North Rhine-Westphalia, Germany

**Keywords:** basic need satisfaction, biology education, motivation, organismic integration theory, perceived pressure, self-determination theory

## Abstract

Within self-determination theory, different operationalizations of motivation co-exist. Person-centered approaches, such as Latent Profile Analysis, are employed to investigate the nuances of motivation and its association with other learning variables. This is particularly advantageous, as profile analyses acknowledge that different forms of motivational regulation are not mutually exclusive and can co-occur within individuals. This study explores different motivational profiles among German upper-secondary level biology students (*N* = 1,419, 17.14 ± 1.48 years, 65% female) and their relationship with perceived basic need satisfaction and pressure. Despite the importance of perceived pressure in determining the quality of experience, few studies have focused on the student perspective. Using Latent Profile Analysis, four motivational profiles were identified: low motivation, average motivation, moderately self-determined motivation, and self-determined motivation. Follow-up analysis of distinct variables indicate a trend that higher proportions of self-determined regulations within a profile were associated with greater perceived need satisfaction and lower pressure. However, students in the average motivation profile reported more perceived pressure without simultaneously reporting lower need satisfaction. Additionally, not the average but the low motivation students reported the lowest levels of perceived autonomy, competence, and relatedness. Thus, it is not the presence of controlled but the absence of self-determined qualities that appears to be associated with low levels of basic need satisfaction.

## 1 Introduction

The natural sciences play a crucial role in societies, contributing to their functioning, progress, and innovation, as well as influencing individual lifestyles. Understanding the natural sciences, developing scientific thinking skills, and mastering the respective scientific tools are essential for individual decision-making and (democratic) participation (Schiepe-Tiska et al., 2016). Therefore, schools are obliged to provide adolescents with various opportunities to acquire *scientific literacy* to cope with these challenges (see e.g., Kultusministerkonferenz, 2020; National Research Council, 1996). Although the scientific literacy approach is rather broad and requires the collaboration of different disciplines (see STEM education), we focused on biology in the current study. The immanent and special importance of this subject and its consequences for one's life are evident in major phenomena such as the corona pandemic or global climate change. Furthermore, the Fridays for Future movement—a global youth-led movement advocating for urgent action on climate change through school strikes and public demonstrations in different countries—shows the relevance of biological issues for young individuals. This is reflected in students perceiving biology, particularly topics related to human biology, as more relevant than the physical sciences (Osborne et al., 2003, p. 1061). Thus, the preconditions of successful *biology* learning are of timeless significance to equip students with skills to cope with such ongoing challenges.

Investigating learning and its preconditions as well as potential threats is a key issue of self-determination theory (e.g., Howard et al., 2021; Linnenbrink-Garcia and Patall, 2016; Ryan and Deci, 2020; Schunk et al., 2014; Wentzel and Miele, 2016). Schools place various demands on their learners, which are, especially within a compulsory school system, mostly of a controlling nature and can go against individual endeavors (Niemiec and Ryan, 2009; Ryan and Deci, 2017). According to SDT, successful and in-depth learning depends on inner involvement and self-determined motivational qualities (Ryan and Deci, 2017). The cross-national relevance of students' motivation as a meaningful determinant of school performance has also been confirmed by international comparative PISA studies (Organization for Economic Co-operation Development, 2024). SDT is a useful framework and has already been successfully applied to biology didactics (see e.g., biology-specific German short-scale of the Intrinsic Motivation Inventory, Wilde et al., 2009; see also Killermann et al., 2024). From a theoretical point of view, motivation is a multidimensional construct with different qualities (Ryan and Deci, 2017). Because of its differentiated conceptualization and dynamic approach, this framework provides an especially insightful basis for both biology education research and classroom practice.

According to Ryan and Deci (2017), motivation can be described in both a quantitative and a qualitative way. The quantitative dimension is determined by the strength or amount of motivation. The second dimension refers to qualitatively different motivation types (Ryan and Deci, 2017). At a superordinate level, intrinsic and extrinsic motivation are distinguished. *Intrinsically motivated actions* are spontaneous and performed out of enjoyment, pleasure, and interest. These activities are initiated and maintained by the individual for their own sake, and are thus perceived as self-determined (Ryan and Deci, 2017). *Extrinsically motivated actions*, on the other hand, are directed toward the achievement of goals and rewards or the avoidance of punishment. Accordingly, they are driven by a purpose that can be separated from the activity itself (Ryan and Deci, 2017). However, several motivational regulation types can be subordinated under the category of extrinsic motivation (Howard et al., 2021; Ryan and Deci, 2017). The type of external regulation that is predominantly perceived as controlled is *external regulation*. Actions based on external regulation are only initiated or maintained as long as the individual expects a direct reward or a punishment (Ryan and Deci, 2017). On the other hand, *introjected regulated actions* are based upon internal pressures and the wish to comply with social norms and receive affirmation from others (Ryan and Deci, 2017). Thus, this regulation is connected to a very low level of perceived self-determination and volition (Ryan and Deci, 2020). *Identified regulation* is the first regulation type of extrinsic motivation, which is accompanied by self-determined qualities of experience (Ryan and Deci, 2020). If an individual identifies with the social norms underlying an action, they initiate and maintain the activity because they feel it is important to them or their future endeavors. However, the action is only maintained as long as it does not compete with other wishes and demands that are more important for the individual (Ryan and Deci, 2017, 2020). This is no longer the case when it comes to *integrated regulated actions*. Here, the social norms and requirements underlying the external demands have been integrated into the individual's self-concept (Ryan and Deci, 2017). Due to this comprehensive internalization, an individual not only acts to meet demands but also because it is personally meaningful and a congruent part of their identity. However, all these regulations imply a striving to be active, to avoid or achieve goals or a state of experiences, or are performed for their own sake, thus they are intentional. In contrast, *amotivated* behaviors are characterized by a lack of motivational regulation (Ryan and Deci, 2017; Vallerand and Ratelle, 2002). All the intentional regulation types can be conceptualized along a hypothetical continuum, namely the self-determination continuum (Ryan and Deci, 2002, 2017; Howard et al., 2021). On this spectrum, all regulations are organized according to their perceived degree of self-determination: intrinsic regulation represents the prototype of fully self-determined behavior, whereas external regulation lies at the opposite end, characterized by low or absent self-determination and high perceived control (Ryan and Deci, 2017; see also Howard et al., 2021; Vallerand and Ratelle, 2002).

However, the aforementioned regulations are not mutually exclusive and may coexist simultaneously within an individual (Chemolli and Gagné, 2014; Ryan and Deci, 2017; Sheldon et al., 2017). Thus, person-centered approaches such as latent profile, class, and transition analyses can be used to consider multidimensionality (Emm-Collison et al., 2020; Geiser et al., 2014; Litalien et al., 2019; Wang et al., 2017). Hereafter, we refer to latent profile analyses (LPA) in particular as one method to explore and characterize hidden latent groups and examine intrapersonal mechanisms and behavioral patterns (Ferguson et al., 2020; Spurk et al., 2020). This is beneficial because it “allows a more detailed examination of the additive or interactive effects of autonomous and controlled motivation in optimal learning” (Vansteenkiste et al., 2009, p. 673). In this context, several studies have revealed different **motivational profiles** consisting of different patterns of the motivational regulations (Emm-Collison et al., 2020; Geiser et al., 2014; Litalien et al., 2019; Petit et al., 2023; Šakan et al., 2024; Sheldon et al., 2017; Tóth-Király et al., 2022; Vansteenkiste et al., 2009; Wang et al., 2016, 2022). Although all these studies emphasize that there are different motivational profiles, they vary in terms of their contexts and their operationalization of motivation. Thus, there is currently no consistent profile characterization. Despite these different operationalizations, some SDT-specific studies based on clustering techniques show several meaningful similarities regarding the reported motivational profiles. The extracted categories and some example studies to which we particularly refer are listed in Table 1.

**Table 1:** Overview of example studies and the profile categories derived from them.

**Superordinate profile characterization**	**Profiles in the respective studies**	**Reference**
Relatively high self-determined and low controlled regulation values	Class 2 and class 3	Geiser et al., 2014
	“The good quality profile”	Vansteenkiste et al., 2009
	Type 3	Wang et al., 2016
	“Self-determined motivated”	Tóth-Király et al., 2022
	“Highly autonomous”	Bechter et al., 2018
	Profile 1	Sheldon et al., 2017
Constantly relatively high values regarding all regulations	Class 4 and class 6	Geiser et al., 2014
	“High quantity motivation”	Vansteenkiste et al., 2009
	Profile 4	Sheldon et al., 2017
	“Strongly motivated”	Tóth-Király et al., 2022
	“Mixed motivation”	Bechter et al., 2018
	Profile 3 (multifaceted)	Litalien et al., 2019
Constantly relatively low values regarding all regulations	Class 4 and class 6	Geiser et al., 2014
	“Low quantity motivation”	Vansteenkiste et al., 2009
	Type 4	Wang et al., 2016
	Profile 5	Sheldon et al., 2017
	“Amotivated”	Bechter et al., 2018
	“Weakly motivated”	Tóth-Király et al., 2022
Relatively high controlled regulations and low self-determined regulations	“Poor quantity motivation”	Vansteenkiste et al., 2009
	Type 2	Wang et al., 2016
	Profile 2 (controlled)	Litalien et al., 2019
	“Moderately controlled”	Bechter et al., 2018

This table focuses on the similarities between the respective studies and does not provide a complete overview about all reported profiles.

In summary, most studies describe at least one type that stands out with high values of self-determined motivational regulations such as intrinsic and identified regulation, and at the same time quite low values of controlled regulations. Moreover, studies reported a profile in which students show either relatively high or low values regarding (nearly) all motivational regulation types. Geiser et al. (2014) found that a profile with low levels of all regulations is associated with high values of amotivation. Furthermore, most studies describe at least one type that is characterized by a relatively low level of autonomous regulations and simultaneously relatively high values regarding controlled regulation types.

Several studies support the assumption of different motivational profiles. However, a study by Geiser et al. (2014) showed that the sub-populations from different countries differ regarding their motivational profile distribution and answer tendencies. Therefore, it is crucial to replicate and further investigate motivational profiles amongst different countries and contexts. For example, Geiser et al. (2014) investigated such motivational patterns in the context of volunteering. In terms of school learning, prior studies often focused on academic motivation in general (Litalien et al., 2019; Marcin et al., 2020; Tóth-Király et al., 2022; Vansteenkiste et al., 2009). Although there are some subject-specific studies [see e.g., physical education (Bechter et al., 2018; Emm-Collison et al., 2020; Wang et al., 2016) or mathematics (Wang et al., 2017), or reading (Wang et al., 2022)], to our knowledge, the motivational profiles have not yet been investigated regarding the subject of biology.

In summary, the existence of different motivational profiles is supported by studies from other contexts and can therefore also be assumed in the context of biology education. As there have been no specific investigations regarding biology education to date, we chose an exploratory approach in the form of an explorative latent profile analysis (see Section 2.3) to examine the following hypothesis:

H1. Latent Profile Analysis reveals specific motivational profiles with quantitatively and qualitatively distinct configurations (i.e., level and shape differences) of students' *intrinsic, identified, introjected*, and *external regulation*.

Because motivation plays a key role in students' learning success as well as positive psychological functioning, it should be considered in association with other learning variables. One particularly important association of students' motivation is the perceived amount of basic psychological need satisfaction (Bechter et al., 2018; Kaiser et al., 2020; Ryan and Deci, 2017; Vallerand and Ratelle, 2002; Vansteenkiste et al., 2009). According to SDT (Ryan and Deci, 2017), there are three equally important and universal **basic psychological needs** for autonomy, competence, and relatedness, which individuals constantly strive to satisfy. The need for autonomy refers to individuals' endeavor to act voluntarily and without external pressure (Reeve, 2018; Ryan and Deci, 2017). Moreover, individuals perceive themselves as origins of their behavior as well as choices when acting autonomously (Reeve, 2018; Ryan and Deci, 2017). The second need for competence describes individuals' striving to perceive their effectiveness and develop their skills in the interaction with the environment (Reeve, 2018; Ryan and Deci, 2017). Lastly, the need for relatedness refers to an individual's wish to interact with significant others and belong to social communities (Reeve, 2018; Ryan and Deci, 2017).

SDT (Ryan and Deci, 2017, 2020) postulates an interdependent relationship between basic need satisfaction and motivation. If individuals perceive themselves as autonomous, competent, and related to others, they are more likely to show self-determined qualities of motivation and less controlled forms thereof (Ryan and Deci, 2017; see also Kaiser et al., 2020; Soenens and Vansteenkiste, 2005; Vansteenkiste et al., 2005). Using profile analysis, (Bechter et al. 2018) found “greater need satisfaction predicted membership of more autonomous profiles” (p. 206). However, the frustration of these psychological needs may encourage heteronomous regulation (Bechter et al., 2018) and may be associated with feelings of anxiety and anger (Assor et al., 2005) as well as alienated learning (Marcin et al., 2020). In this context, the three basic needs can show differing predictive effects on the respective motivational regulations (Kaiser et al., 2020) and profiles (Bechter et al., 2018). At the same time, motivation and need satisfaction operate reciprocally; for instance, intrinsic and identified regulations can further enhance feelings of autonomy, competence, and relatedness (Ryan and Deci, 2017). Due to this essential relationship, we compared the respective motivational profiles in terms of students' perceived basic need satisfaction. Accordingly, we tested the following non-directional hypothesis:

H2. Students of the respective profiles differ regarding their perceived *autonomy* (H2a), *competence* (H2b), and *relatedness* (H2c).

Another important factor that determines students' emotional quality of experience is **perceived pressure**. The impact of teachers' perceived job pressure is of special interest within SDT (see e.g., Bartholomew et al., 2014; Martinek, 2019; Pelletier et al., 2002; Pelletier and Sharp, 2009; Ryan and Deci, 2017; Taylor et al., 2008). However, there is currently only a small number of studies concerning the perspective of students in their respective school subjects (Kaiser et al., 2021; Martinek and Carmignola, 2020). Perceived pressure refers to the subjective evaluation of school-related demands that make students *feel pressured*, describing the individual experience of these demands in relation to personal resources and interpretations (Kaiser et al., 2021). Kaiser et al. (2021) evaluated students' perceived pressure in biology lessons and found three distinct dimensions: *time pressure, pressure by performance requirements*, and *pressure by teachers*. In particular, teaching behavior (autonomy-supportive vs. controlling) and teachers' handling of performance assessments (e.g., as informative feedback vs. extrinsic motivator for disciplinary issues) are key elements of SDT research (see e.g., Assor et al., 2005; Großmann and Wilde, 2020; Jang et al., 2010; Moreno-Murcia et al., 2018). Thus, teachers may cause feelings of pressure within students. Furthermore, perceived pressure may act as an external or internal (e.g., in the case of introjected regulations) motivator (Ryan and Deci, 2002, 2017). While perceived pressure has been investigated in terms of perceived basic need satisfaction using structural equation modeling (regarding students, see e.g., Kaiser et al., 2021; regarding teachers, see e.g., Bartholomew et al., 2014; Martinek, 2012), to our knowledge, there are currently no studies focusing on the interdependencies between students' perceived pressure and their motivation. In the current study, we addressed this desideratum and tested the following non-directional hypotheses:

H3. Students of the respective profiles differ regarding their perceived *time pressure* (H3a), *performance pressure* (H3b), and *pressure by their teacher* (H4c).

## 2 Methods

### 2.1 Design and participants

We conducted a cross-sectional study to question students with a one-time paper-pencil survey about their biology education. The sample consisted of 1,419 students (*M*_*age*_ = 17.14 years, *SD*_*age*_ = 1.48 years, 65% female) from 10 different secondary schools from the federal state of North Rhine-Westphalia in Germany. Participants were recruited from schools in the surrounding area using a convenience sampling approach. The slight imbalance of girls and boys was a result of the fact that some of the classes that participated belong to a vocational school with the compulsory focus on “health and social care” in which girls were overrepresented. In general, only upper-secondary level students from grades 10 to 13 were questioned. These students came from 69 biology classes (66% basic and 34% advanced courses) and were questioned during their biology lessons by university members. Ethical aspects were constantly considered throughout the planning, implementation, and evaluation of the study. Approval to conduct the study was always obtained from the school principal or a designated representative. Students were carefully informed about the study's objectives and methodological approach by a standardized procedure, and verbal consent was obtained. Participation was voluntary and there were no disadvantages for the students if they did not complete the survey. At no point, teachers or school administrators were involved in data collection or evaluation. Furthermore, all data were fully anonymized, preventing any identification of individuals or specific classes.

### 2.2 Measures

We used a quantitative survey with standardized closed-ended items. The students were asked to rate some statements on their biology lessons on a five-level verbal rating scale from “not true” (coded with 0) to “absolutely true” (coded with 4). To assess their motivation, we used Thomas and Müller's (2016) *scales measuring motivational regulation for learning* (SMR_L), which is a translated and validated German version of the self-regulation questionnaire (SRQ, Ryan and Connell, 1989). This measurement operationalized students' motivational regulation with the four subscales *intrinsic* (3 items)*, identified* (3 items)*, introjected* (4 items), and *external regulation* (3 items). In contrast to the regulations outlined above (see chapter Introduction), the SMR_L measure does not assess all motivational regulations. First, it only captures intentional actions; accordingly, amotivation-defined as the absence of intentional behavior regulation (Ryan and Deci, 2017) is not included. Second, integrated motivational regulation is also not measured with this instrument, as determining this type of regulation would require information about the test subject's values and attitudes (Thomas and Müller, 2016).

Furthermore, to assess their *perceived basic need satisfaction*, we relied on a translated version of the work-related satisfaction scale (W-BNS, Van den Broeck et al., 2010) that has been adapted to the school context (see e.g., Kaiser et al., 2020; Kirchhoff et al., 2023). This self-report measure assesses students' perception of *autonomy* (5 items), *competence* (4 items), and *relatedness* (3 items). As a negative dimension of emotional quality of experience, we evaluated the students' perceived pressure in biology education according to Kaiser et al. (2021). The scale consisted of three subscales evaluating students' *time pressure* (4 items)*, performance pressure* (5 items), and *pressure by teachers* (4 items).

### 2.3 Statistical procedure

On a general level, the statistical procedure can be distinguished in preliminary analyses, model retention decision (LPA), and follow-up analyses. We used *IBM SPSS Statistics 27* for data management and basic analyses such as calculating means, checking for outliers, and assessing the internal consistency for each subscale. For the advanced analyses, we used the *MPlus Base Program* for confirmatory factor analysis and the *Mixture Add-On* (Muthén and Muthén, 2017) for LPA.

#### 2.3.1 Preliminary analysis

We used Latent Profile Analysis (LPA) to identify different motivational profiles regarding biology education amongst German students from the upper secondary level and their relationship to auxiliary variables (Asparouhov and Muthén, 2014), namely their basic need satisfaction, perceived pressure as well as demographic characteristics (sex, age, and gender). As classification variables for the LPA, we used the scale means of the four subscales of motivational regulation (intrinsic, identified, introjected, and external regulation). In this context, a confirmatory factor analysis was conducted to control if the sample means represent the assumed factors. As chi-square testing is quite sensible to large sample sizes, we used the CFI, RMSEA, and SRMR as additional parameters to estimate the quality of the measurement model (Hooper et al., 2008). Furthermore, we determined the Cronbach's alpha values to test the internal consistency of all subscales.

#### 2.3.2 Model retention decision

Based on these preliminary analyses, we conducted an explorative LPA, by calculating several iterative LPA models to determine a suitable model with an appropriate number of profiles (Ferguson et al., 2020). To reduce the risk of local likelihood-maxima, we increased the number of starting values to 1,000 and the number of iterations to 500 (Geiser, 2010). Furthermore, we used robust measures (MRL) for the analyses to account for deviations from normality within groups (Muthén and Muthén, 2017). As information criteria parameters, we followed Nylund et al. (2007) recommendations and used the Bayesian Information Criterion (BIC), the adjusted Bayesian Information Criterion (aBIC), and the Akaike's Information Criterion (AIK) (see also Ferguson et al., 2020; Geiser, 2010). We also used the entropy measure as “a statistical measure of uncertainty” to describe the model fit (Ferguson et al., 2020, p. 3). As an additional descriptive model fit indicator, we examined the average classification probability for each scale (Geiser, 2010). Furthermore, we substantiated our analysis with statistical testing methods such as the Vuong-Lo-Mendell-Rubin (VLMR) test (Geiser, 2010; Nylund et al., 2007). This method is based on a comparison of the respective profile model (*k*) with the previous one (*k*-1). Accordingly, a significant *p*-value indicates that the respective *k*-profile provides a significantly better model than the previous one (Geiser, 2010). However, these methods are also quite sensitive to big sample sizes, and it is possible to reduce, for example, the information criterion values by increasing the number of profiles (Marsh et al., 2009). This does not often lead to qualitatively better profile solutions (Ferguson et al., 2020; Marsh et al., 2009). Therefore, the respective profile solution should be viewed critically concerning the theory and previous research findings (Ferguson et al., 2020) and the favored model should include as few profiles as possible (*principle of parsimony*; Geiser, 2010; see also Vandekerckhove et al., 2015).

#### 2.3.3 Follow-up analysis

To investigate the “relationships and differences between latent groups” (Ferguson and Hull, 2019, p. 461), we also examined the motivational profiles in relation to different *auxiliary variables*. As the modeling process might become distorted by covariates when using a single-step-mixture model (Asparouhov and Muthén, 2014; Bakk and Vermunt, 2016; Ferguson et al., 2020), we followed a three-step approach suggested by Asparouhov and Muthén (2014): (1) enumeration of the latent profiles (described above under Model Retention Decision), (2) estimation of class classification error probabilities, and (3) analysis of auxiliary variables. Applying the BCH method, we considered the individual measurement errors of the latent class variable in relation to auxiliary variables in two ways, depending on the assumed relationship between profile membership and each auxiliary variable (Asparouhov and Muthén, 2014; Bakk and Vermunt, 2016; Ferguson et al., 2020).

First, we used profile membership to predict differences in perceived basic need satisfaction (*autonomy, competence*, and *relatedness*) as well as perceived pressure (*time pressure, performance pressure*, and *pressure by the teacher*). To do so, the BCH approach was implemented using the automatic procedure in Mplus (see “auxiliary” option in Mplus, Muthén and Muthén, 2017, Chapter 7). In this approach, differences between profiles are evaluated using Wald χ^2^ tests. Therefore, overall effect sizes are reported as Cramér's *V* and pairwise differences are evaluated as standardized mean differences.

Second, we controlled for the possible effects of *background variables* (individual characteristics such as age, sex, and course level) by analyzing these as predictors of the respective class assignment using the manual three-step BCH approach (Asparouhov and Muthén, 2014; Ferguson et al., 2020).

For the follow-up analysis, fifteen participants had to be excluded due to missing data (*n* = 1,404).

## 3 Results

### 3.1 Preliminary analyses

First, we evaluated the measurement model of the scales assessing students' motivational regulation during learning. Despite the significant chi-square test (χ^2 =^ 5968.78, *p* < 0.001) parameters indicated an acceptable model fit (Geiser, 2010; Hooper et al., 2008). Furthermore, the subscales *intrinsic* (α = 0.89)*, identified* (α = 0.76)*, introjected* (α = 0.77), and *external regulation* (α = 0.69) showed acceptable internal consistencies. Accordingly, the scale means seem to be suitable to represent the theoretically and empirically (Thomas and Müller, 2016) assumed factors and are thus appropriate model indicator variables for the LPA.

Additionally, we checked the reliability of the variables that were used for the follow-up analysis. The measurement models of the scales assessing the perceived basic need satisfaction (χ^2 =^ 5831.99, *df* = 66, *p* < 0.001; CFI = 0.95; RMSEA = 0.06; SRMR = 0.04) and the internal consistencies (*autonomy* α = 0.78, *competence* α = 0.89, and *relatedness* α = 0.77) indicate an acceptable fit. Lastly, we evaluated the measurement assessing students' perceived pressure. Here again, the CFA (χ^2 =^ 435.79, *df* = 66, *p* < 0.001; CFI = 0.92; RMSEA = 0.07; SRMR = 0.05) as well as the internal consistencies of all subscales [*time pressure* (α=0.75), *performance pressure* (α=0.74), and *pressure by teachers* (α=0.80)] were acceptable.

Bivariate correlations between all scales are available in the Online Appendix (see Table 5).

### 3.2 Model retention decision

To identify the appropriate number of profiles, we compared the model fit of the respective class solutions with each other. A summary of all models is presented in Table 2.

**Table 2:** Latent profile analysis model fit summary.

**Model**	**Log-likelihood**	**Smallest class % *(n)***	**Information criteria**				**VLMR Test**			**Class-assignment probability**	
			**AIC**	**BIC**	**aBIC**	**Entropy**	**VLMR**	**Meaning**	***p-*value**	**min**	**max**
**2 profiles**	−6887.97	41.08% (483)	13801.94	13870.00	13828.69	0.71	−7275.74	2 vs. 1	< 0.001	0.89	0.93
**3 profiles**	−6699.51	23.33% (331)	13435.02	13529.25	13472.07	0.76	−6887.97	3 vs. 2	< 0.001	0.86	0.90
**4 profiles**	–**6603.87**	**6.34% (90)**	**13253.74**	**13374.14**	**13301.08**	**0.78**	–**6699.51**	**4 vs. 3**	**>0.05**	**0.80**	**0.89**
**5 profiles**	−6504.36	4.30% (61)	13064.73	13211.31	13122.36	0.80	−6603.87	5 vs. 4	0.168	0.79	0.90
**6 profiles**	−6451.94	3.24% (46)	12969.87	13142.62	13037.80	0.81	−6504.36	6 vs. 5	>0.05	0.77	0.90

*n* = 1,390; AIC, Akaike's information criterion; (a)BIC, (adjusted) Bayesian information criterion; VLMR, Voung-Lo-Mendell Ruben Test. The favored class model is marked in bold print.

The comparison of the respective class-models favors the four-profile model. In the following section, the model retention decision is presented stepwise. On the one hand, the information criteria kept decreasing, indicating that more than six profiles were needed. However, as mentioned in the previous section, LPA might be affected by the sample size, and better model fit can be produced by increasing the profile number (Marsh et al., 2009). However, this is often connected to a loss of distinction between the profiles and small sample sizes in the profiles (Ferguson et al., 2020, p. 460). The VLMR test showed that the five-profile solution did not provide significantly more information than the previous one. Furthermore, a comparison of the four- and six-profile solutions showed that the average class-assignment probability decreases when increasing the profile number to six. This led to a lower distinctiveness and reliability of the profiles. According to Geiser (2010), average class-assignment probabilities of 0.80 and above are desirable to enable a clear classification and decrease the respective error probability. In addition, the six-profile model led to a relatively small group (only 43) of students (3%) that is more likely to be neither meaningful nor representative (Ferguson and Hull, 2019). Lastly and most importantly, the four-profile model was in line with pre-existing studies, while the models with more profiles led to similar profile patterns, which only differed regarding the quantity but did not lead to qualitatively distinct profiles. Therefore, and because the more parsimonious option should always be preferred (*principle of parsimony*; see e.g., Geiser et al., 2014; Ferguson et al., 2020), we decided on the four-profile model as the best solution.

### 3.3 Characterization of the motivational profiles

**Figure 1:**
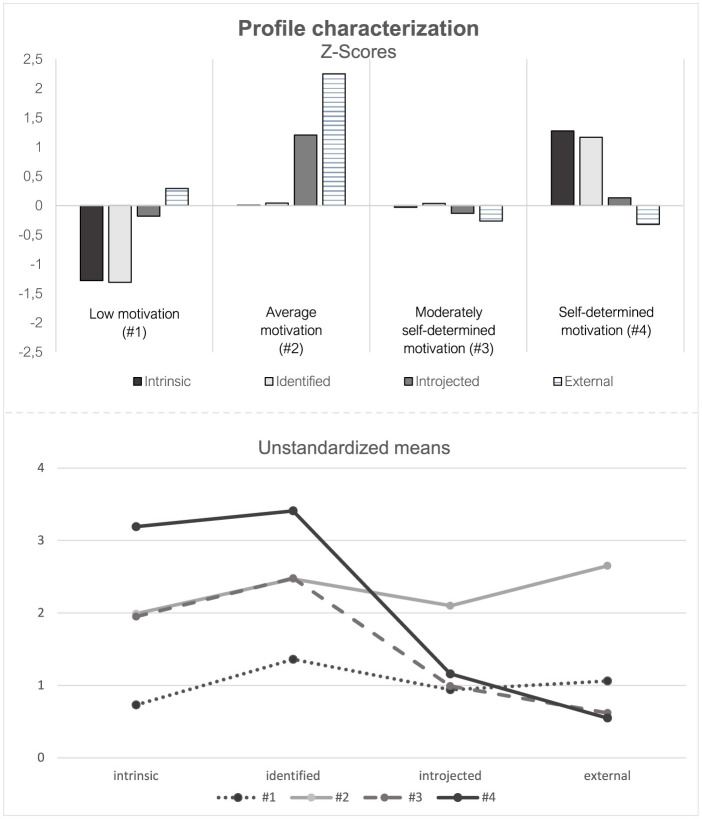
Characterization of the 4-profile model in terms of the model indicators intrinsic, identified, introjected, and external regulation for Z-standardized and unstandardized means.

The respective motivational profiles are illustrated in Figure 1. The standard deviations of all model indicators are available in the Online Appendix (Table 6).

Profiles are named and mainly described based on raw scores (see lower part of Figure 1). Three hundred five students (22%) were classified into Profile 1, characterized by a distinct response pattern with consistently low levels across both self-determined (intrinsic and identified) and controlled (introjected and external) motivational regulations based on a response scale from 0 to 4. Accordingly, this group is referred to as students with “**low motivation**.” The z-scores (see upper part of Figure 1) additionally indicate that self-determined regulations are below average while the controlled regulations are approximately at the sample mean. Profile 2 included 90 members (6%) and is the smallest of all profiles. These students reported average levels of all regulations around the scales' midpoint. Therefore, we named this profile “**average motivation**.” However, considering z-scores also allows for a more detailed illustration. Students from this profile stand out with, above average high values of introjected and especially external regulation. In other words, within our sample, the relatively high levels of the controlled regulations are indicative for this profile in contrast to the other profiles. However, the quantity of the self-determined regulations corresponded to the overall mean of the sample (*M*_intrinsic_ = 1.99, *SD*_intrinsic_ = 0.57; *M*_identified_ = 2.47, *SD*_identified_ = 0.76). Nearly half of all students belonged to profile 3 (*n* = 688; 49%), which is characterized by moderately high levels of intrinsic and identified regulation as per scale midpoint. At the same time, the students reported a low level of introjected and external regulations. In reference to Tóth-Király et al. (2022), this profile was named “**moderately self-determined motivation**.” Profile 4 was predominated by self-determined qualities and showed a low level of introjected and external regulation. This pattern is evident in both the raw and the z-standardized scores. As a result, it was referred to as “**self-determined motivation**”. In total, 336 students (23%) showed this specific motivational pattern. Overall, it is noticeable that the levels of the controlled qualities introjected (*M* = 1.09; *SD* = 0.89) and external regulation (*M* = 0.83; *SD* = 0.84) were relatively low compared to intrinsic (*M* = 1.98; *SD* = 1.01) and especially identified (*M* = 2.45; *SD* = 0.86) regulation in our sample (see Online Appendix, Table 6).

### 3.4 Follow-up analysis

**Figure 2:**
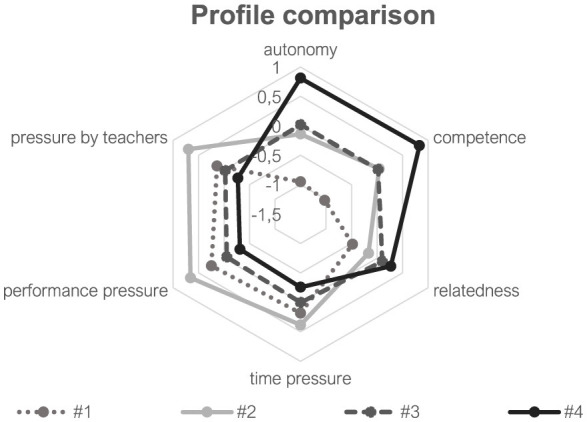
Comparison of the four motivational profiles regarding perceived basic need satisfaction (autonomy, competence, and relatedness) and perceived pressure (time pressure, performance pressure, and pressure by teachers). Illustrated are the Z-Scores. Profile names: 1 = low motivation; 2 = average motivation; 3 = moderately self-determined motivation; 4 = self-determined motivation.

Based on the model retention process, a follow-up analysis was conducted to specify the respective profiles in terms of students' perceived basic need satisfaction and pressure. The specific results are illustrated in Figure 2.

Furthermore, we investigated whether the students' demographic characteristics (sex, age, and course level) predicted their profile assignment. The analysis revealed varying relationships between the motivational profiles and students' perceived amount of basic need satisfaction and perceived pressure. For a detailed overview, see Figure 2 and Table 3.

**Table 3:** Differences between the respective profiles.

**Subscale**	**Motivational profiles (z-standardized means)**				**Profile comparisons (mean differences)**					
**Perceived**	**1**	**2**	**3**	**4**	**1-2**	**1-3**	**1-4**	**2-3**	**2-4**	**3-4**
Autonomy	−0.95	−0.14	0.02	0.81	−0.81^***^	−0.97^***^	−1.76^***^	−0.16	−0.95^***^	−0.79^***^
Competence	−1.03	0.04	0.02	0.83	−1.07^***^	−1.05^***^	−1.86^***^	0.02	−0.80^***^	−0.81^***^
Relatedness	−0.48	−0.17	0.10	0.27	−0.31^*^	−0.58^***^	−0.74^***^	−0.27	−0.44^*^	−0.17^**^
Time pressure	0.18	0.38	0.00	−0.26	−0.20	0.18^*^	0.44^***^	0.38^***^	0.64^***^	0.26^***^
Performance pressure	0.25	0.66	−0.05	−0.31	−0.41^**^	0.30^***^	0.56^***^	0.71^***^	0.97^**^	0.26^**^
Pressure by teachers	0.14	0.70	−0.02	−0.27	−0.56^***^	0.16	0.41^***^	0.72^***^	0.97^**^	0.25^**^

Presented are the difference values of the respective profile comparison. Continuous variables were z-standardized and differences can thus be interpreted according to Cohen's d. Differences between the respective groups were examined through equality tests of means across classes using the BCH procedure. Significant levels: ^*^ = *p* < 0.050, ^**^ = *p* < 0.010, ^***^ = *p* < 0.001. Profile names: 1 = low motivation; 2 = average motivation; 3 = moderately self-determined motivation; 4 = self-determined motivation.

#### 3.4.1 Perceived basic need satisfaction

Follow-up analysis revealed significant overall effects in terms of students' perceived *autonomy* (χ^2^ = 494.595, *p* < 0.001; Cramér's *V* = 0.34), *competence* (χ^2^ = 613.809, *p* < 0.001; Cramér's *V* = 0.38), and *relatedness* (χ^2^ = 78.00, *p* < 0.001; Cramér's *V* = 0.14). We used Cramér's *V* (3 *df* ) for estimating effect sizes where *V* > 0.06 represents a small, >0.17 a medium, and >0.29 a large effect (Cohen, 1988). Cramér's *V* indicates large effects regarding the scales perceived autonomy (34% explained variance) and competence (38% explained variance). By contrast, we found a small effect regarding the students' perceived relatedness (14% explained variance). In the following section, we highlight some characteristic findings.

First, the *self-determined motivated* (#4) students reported consequently higher scores of perceived autonomy, competence, and relatedness compared to all other groups. Regarding autonomy and competence, we found relatively large differences. Accordingly, the self-determined motivated profile was accompanied with positive emotional qualities of experience. By contrast, the *low motivation* (#1) profile was accompanied by the lowest values of perceived autonomy and competence compared to all other profiles. Regarding relatedness, we found small differences in favor of the students from profiles 3 (*moderately self-determined motivation*) and 4 (*self-determined motivation*). Furthermore, we did not find differences between the *average motivation* (#2) and *moderately self-determined motivation* (#3) group in terms of the perceived basic need satisfaction. Accordingly, profiles 2 and 3 showed similar patterns in terms of the positive qualities of experience (see Table 3).

#### 3.4.2 Perceived pressure

In complement to basic need satisfaction, we evaluated the associations between the profiles and the students' perceived pressure. In this context, we also found significant overall effects in terms of *time pressure* (χ^2^ = 35.037, *p* < 0.001; Cramér's *V* = 0.09), *performance pressure* (χ^2^ = 74.446, *p* < 0.001; Cramér's *V* = 0.13), as well as *pressure by teachers* (χ^2^ = 53.028, *p* < 0.001; Cramér's *V* = 0.11). In contrast to the perceived basic need satisfaction, Cramér's *V* indicated only small effects (Cohen, 1988). The specific means (z-scores) and the respective profile differences are listed in Table 3, while we highlight only some characteristic findings in the following section.

Compared to all other profiles, the students from the *self-determined motivation* (#4) profile reported the lowest amount of time pressure as well as the lowest pressure by performance requirements and the teacher (see Table 3). Furthermore, the *average motivation* (#2) students reported higher scores regarding perceived performance pressure and pressure by their teachers than those from all other profiles. However, regarding perceived time pressure, these associations were either not significant (see comparison #1 vs. #2) or the z-standardized differences were mostly smaller than 0.5 (except #2 vs. #4) and thus indicated rather small effects (Cohen, 1988). Furthermore, we would like to highlight the specific differences in terms of the *average motivation* (#2) and the *moderately self-determined motivation* (#3) profiles. While both profiles did not differ regarding their positive qualities (i.e., need satisfaction), we found differences in terms of all three pressure dimensions in favor of profile 3.

#### 3.4.3 Background variables

Lastly, we controlled for potentially confounding effects of individual characteristics such as gender, age, and course level assignment. The results of the logistic regressions according to the manual BCH approach are presented in Table 4. In accordance with Chen et al. (2010), we focused on differences with at least a small effect size (ORs >1.68 and ORs < 0.60; see p. 862).

**Table 4:** Covariates predicting latent profile membership.

	**Profile 1 vs. 2** ^*^			**Profile 1 vs. 3** ^*^			**Profile 1 vs. 4** ^*^			**Profile 3 vs. 2** ^*^			**Profile 2 vs. 4** ^*^			**Profile 3 vs. 4** ^*^		
**Predictor**	**OR**	**SE**	**Sig**.	**OR**	**SE**	**Sig**.	**OR**	**SE**	**Sig**.	**OR**	**SE**	**Sig**.	**OR**	**SE**	**Sig**.	**OR**	**SE**	**Sig**.
**Sex** (0 = female, 1 = male)	1.46	0.43	0.283	**2.24**	**0.41**	**< 0.010**	**2.47**	**0.49**	**< 0.010**	0.65	0.19	0.064	1.69	0.50	0.170	1.10	0.21	0.620
**Age**	1.38	0.17	< 0.050	1.08	0.06	0.168	1.26	0.12	< 0.050	1.28	0.18	0.066	0.91	0.12	0.468	1.17	0.10	0.077
**Course level** (0 = basic, 1 = advanced)	**0.31**	**0.12**	**< 0.001**	**0.32**	**0.08**	**< 0.001**	**0.11**	**0.03**	**< 0.001**	0.99	0.34	0.969	**0.36**	**0.13**	**< 0.001**	**0.36**	**0.07**	**< 0.001**

Presented are the Odds-Ratios (OR), their standard errors (SE), as well as their significance (Sig.). The respective reference class is marked with ^*^. Significant Odds-Ratios larger 1.68 and smaller 0.60 are displayed in bold (see Chen et al., 2010). Profile names: 1 = low motivation; 2 = average motivation; 3 = moderately self-determined motivation; 4 = self-determined motivation.

With regard to gender, we found that the *low motivation* students (#1) were more likely to be male compared to the students from the *self-determined* (#4) and the *moderately self-determined motivation* (#3) group. In terms of the course level, we found that the students from the *low motivation* (#1) group were less likely to visit an advanced course compared to all other groups. By contrast, the s*elf-determined motivation* (#4) students were more likely to attend an advanced course than the learners from all other profiles.

## 4 Discussion

The current study examined students' motivation regarding biology by using a person-centered approach. As assumed, we found distinct motivational profiles that differed from each other. Furthermore, we found differences between the respective groups (*low motivation, average motivation, moderately self-determined motivation*, and *self-determined motivation*) regarding their perceived basic psychological need satisfaction (H2) as well as their perceived pressure (H3). As these relationships extend the profile characterization, we discuss these profile-specific associations in combination with the respective profiles.

First, we found a *self-determined motivation profile* (#4) that is characterized by a high level of intrinsic and identified regulations. This finding corresponds with previous studies using person-centered approaches, indicating that this pattern represents a “core-profile” (Tóth-Király et al., 2022, p. 154) that can be replicated across different studies despite different conceptualizations (see e.g., Bechter et al., 2018; Geiser et al., 2014; Sheldon et al., 2017; Tapia-Serrano et al., 2024; Wang et al., 2016, 2022). The self-determined motivated profile was accompanied by a high sense of perceived autonomy, competence, and relatedness. At the same time, these positive qualities of experiences were higher in this group than in all other groups. Regarding their *perceived pressure*, our findings drew a coherent picture: The students with a self-determined motivation profile reported the lowest time pressure, pressure by performance requirements, and pressure by the teacher. This profile reflects the most desirable motivational predisposition (Ryan and Deci, 2017; Vansteenkiste et al., 2009). In addition to our findings, studies have also investigated (self-determined) motivational profiles in terms of performance outcomes. As indicated by a study by Tóth-Király et al. (2022), such a profile predominated by self-determined regulations is also accompanied by more engagement in form of vigor, dedication, and absorption. Furthermore, these students report better grades and are less likely to voice drop out intentions (Tóth-Király et al., 2022). This is especially relevant in the context of school learning as schools provide various demands on students, which are mostly of a controlling nature (Reeve and Assor, 2011; Ryan and Deci, 2017).

Against the backdrop of previous studies (see Table 1), we assumed we would find at least one group with relatively low values of all regulations. In our study, the *low motivation profile* (#1) seems to represent this type. However, what stood out was the below-average level of intrinsic and identified regulation, which was, in contrast to the low quantity profile proposed by Vansteenkiste et al. (2009), not accompanied by an unusually low level of controlled regulation. This profile was accompanied by the lowest perception of autonomy and competence. On a general level, the students from this profile tended to report more perceived pressure than the students from the profiles *self-determined motivation* (#4) and *moderately self-determined motivation* (#3), although less than the *average motivation profile* (#2).

Furthermore, SDT research also focused on specifying and understanding the controlled nature of human motivation (Ryan and Deci, 2020). Various person-centered studies found at least one motivational profile, which is predominated by controlled regulations, such as the “poor quality profile” proposed by Vansteenkiste et al. (2009). Thus, we expected to also find one profile which would show high levels of controlled and at the same time relatively low levels of self-determined regulations. However, although we found one profile with above-average controlled regulations that was also accompanied by high perceived pressure, this was not inevitably connected to pronounced low levels of self-determined regulations. Our findings stress that approximately self-determined and controlled qualities are, at least to a certain degree, not mutually exclusive (Chemolli and Gagné, 2014; Ryan and Deci, 2017; Sheldon et al., 2017). Furthermore, this *average motivation profile* was not automatically associated with a low level of basic need satisfaction. By contrast, it was not the *average motivation* (#2) but the *low motivation* (#1) students who reported the lowest perception of autonomy and competence. In their study, Tóth-Király et al. (2022) reported similar findings, where the “strongly motivated profile” also showed clearly higher levels of the external and introjected regulation, though without extraordinarily low levels of self-determined regulations (see #2). In this context, the “strongly motivated” students showed some similarities to the “self-determined motivated” in terms of life satisfaction, low drop-out intentions and grades (Tóth-Király et al., 2022). This suggests that a high level of rather controlled regulations is not necessarily detrimental when combined with relatively high (Tóth-Király et al., 2022) or at average (see #2) levels of self-determined regulations.

In addition, we found the *moderately self-determined motivation profile* (#3), which showed a similar pattern as the *average motivation profile* (#2) regarding the intrinsic and identified regulations. However, while profile 2 stood out with above-average levels of controlled regulations, profile 3 students reported relatively low levels of introjected and external regulations. Nearly half of the questioned students belonged to this profile. Students from both profiles (#2 and #3) do not differ from each other in terms of the positive qualities, as they show similar levels of perceived autonomy, competence, and relatedness. However, in line with the higher levels of controlled regulations, the *average motivation* (#2) students scored higher in all three pressure dimensions than the moderately motivated students.

At a general level, the following tendency can be observed: The higher the amount of self-determined motivational regulations in the profile, the higher the extent of perceived need satisfaction and the lower the perceived pressure. However, a low level of these self-determined qualities, such as in the low motivation profile, is also correlated with lower levels of perceived need satisfaction. Accordingly, our study reproduces one of the basic assumptions of SDT (Ryan and Deci, 2017) and is in line with previous variable-centered (Kaiser et al., 2020; Kusurkar et al., 2013) and person-centered approaches (see e.g., Bechter et al., 2018; Litalien et al., 2019; Tóth-Király et al., 2022; Vansteenkiste et al., 2009). Similarly, the only profile in which the controlled qualities were above average (#2) was also found to be associated with the highest degree of perceived pressure. However, higher controlled regulations, accompanied by a higher sense of pressure, apparently does not inevitably lead to a frustration of the basic psychological needs, for the *average motivation* students (#2) did not report less perceived autonomy, competence, and relatedness than the students grouped into the *moderately self-determined motivation* group (to which nearly half of the students belong to; #3). By contrast, the lowest levels of basic needs were shown by the students from the *low motivation* group (#1). Accordingly, it is not necessarily the sole presence of controlled regulations, but a lack of autonomous qualities that is primarily associated with low levels of perceived autonomy, competence, and relatedness. In the context of emotions, it could be shown that positive and negative qualities are not necessarily mutually exclusive but may occur simultaneously if no quality predominates (Diener and Iran-Nejad, 1986). This highlights the benefits of person-centered analyses, as they reveal more complex interactions and account for the co-occurrence of different qualities. However, these tendencies should be investigated further across different contexts.

### 4.1 Limitations

To summarize, the current study revealed four distinct motivational profiles in the subject of biology and their specific associations to positive (the perceived basic psychological need satisfaction) and negative qualities of experiences (the perceived pressure). Besides these findings, some limitations should be considered. First, it is noticeable that across all profiles, the students reported relatively high values regarding intrinsic and identified regulation in particular and at the same time relatively low levels of the controlled qualities introjected and external regulation. This phenomenon can be observed in several studies using this measurement (see e.g., Kaiser et al., 2020; Thomas and Müller, 2016). In this context, the comparatively lower levels of controlled qualities are often accompanied by slightly lower internal consistencies. Furthermore, this effect can also be observed in studies assessing students' pressure and tension as conceptualized by the Intrinsic Motivation Inventory and its respective adaptions (Wilde et al., 2009). Accordingly, this is not an unusual pattern within the SDT framework. This might be explained on a psychometric as well as a psychological level. First, measurement issues are discussed. Self-reports are a common method to evaluate individuals' attitudes and self-perception. On the other hand, this approach is quite vulnerable to distorted answer tendencies (Döring and Bortz, 2016). Students might not want to admit (even to themselves) that they are driven by undesirable reasons such as to avoid punishment or to outperform classmates. As we questioned mainly students from the upper secondary level biology courses, a rather sensitive stage of their school career where final exams are pending, it can be assumed that the levels of the controlled qualities could have been underestimated.

From a theoretical point of view, the high values regarding self-determined and relatively low values of the controlled scales might be explained by the institutional nature of schools. In Germany, there is a compulsory school system that forces students to go to school and participate in classes even when it is against their wishes and interests. Thus, school inherits a controlling nature regardless of the structure of the respective lessons (Reeve and Assor, 2011). A qualitative study by Nüberlin (2002) showed that many German students do not question the purpose and the relevance of the school, and especially the performance requirements. On the contrary, they report that it is important for themselves and their future career aspirations. This shows that most students show a relatively high internalization level (Ryan and Deci, 2017). This may allow them to perceive a sense of autonomy and volition inside a relatively restrictive system (Ryan and Deci, 2017) and might explain these high values, especially concerning identified regulation. In addition, this effect might be promoted by the nature of biology. Biology is a subject that partly allows to address issues of everyday life (Kultusministerkonferenz, 2020; National Research Council, 1996). Therefore, students perceive it as meaningful and relevant for their personal lives (Osborne et al., 2003). This could facilitate internalization processes.

Furthermore, we would like to highlight some characteristics of the sampling and the German school system. In our study, we investigated students from the upper secondary level who were (at least partly) able to choose if they would like to study biology if they take their final exams in this subject. Therefore, we questioned mainly students who are probably more interested in the subject beforehand (see interest specialization and individual interest; Krapp, 2005), while those who were less interested chose other subjects which are more in line with their interests and strengths. In this context, Wegner and Schmiedebach (2020) found that students in the 12^th^ grade reported significantly more interest regarding biology than students from the lower-secondary level (here Grades 7 and 9). This might also explain the high standard of intrinsic and identified regulations and should be considered in terms of transferability to other subjects.

### 4.2 Conclusion and practical implications

Despite some methodological limitations, our study revealed meaningful motivational profiles in the context of upper-secondary level biology education. Our results indicate that, it was not the presence of controlled motivation but rather the absence of self-determined qualities that was associated with low levels of need satisfaction. However, motivational profiles, especially those with less desirable characteristics (see e.g., profile #1) should not be viewed as unchangeable conditions, as previous research suggests that such profiles can change over time. A study by Emm-Collison et al. (2020) investigated motivational profiles for exercise among the parents of British primary school children. First, this study also confirmed the existence of several motivational profiles. Second, they indicated that self-determined motivational profiles are connected to positive outcomes such as a higher engagement in physical activity. These two aspects are in line with prior research and the findings of the current study. Third, they investigated the development of these profiles over a 5 year period. In this context, they found that “(…) individuals were more likely to move between profiles than to remain in the same one, indicating that motivation for exercise is relatively dynamic” (Emm-Collison et al., 2020, p. 7). However, the self-determined profiles were more stable than those predominated by controlled regulations. Accordingly, practitioners should provide interventions that aim to support (self-determined) motivation, especially for students belonging to the *low motivation profile* (#1), rather than accepting these motivational profiles as natural and fixed conditions.

Applied to biology learning, these findings encourage the striving to foster students' self-determined biology learning and stress the need for a critical view of the subject itself as well as the learning setting in general. First, as a hands-on subject, biology itself provides a wide range of opportunities for interest-driven, inquiry-based, and problem-based learning through the implementation of experiments, explorations, and observations of natural phenomena (Kultusministerkonferenz, 2020). These opportunities may be further extended by field trips that allow students to explore phenomena first-hand and under authentic scientific conditions (see e.g., Kirchhoff et al., 2023). This might support students' perception of autonomy, competence, and relatedness by offering choice, hands-on engagement, and collaborative learning. Second, biology addresses crucial aspects of students' everyday life as well as health and socio-scientific issues which may frame the learning context (see providing a *rationale*; Ryan and Deci, 2017). As many general principles can be explored in various contexts (see *principle of exemplarity;* see e.g., Killermann et al., 2024), teachers have the opportunity to provide students with *real* choices (Reeve, 2018). Katz and Assor (2007) demonstrated that motivation does not result from offering choice *per se*, but primarily from providing choices that are personally relevant, and that are also manageable in complexity and number as well as socially meaningful (see also Reeve, 2018). The exemplary character of the subject biology allows principles such as synaptic transmission and neuronal plasticity (Kultusministerkonferenz, 2020) to be explored in various different contexts. These include the effects of substances such as nitrous oxide, the impact of social media on the brain's reward system, or variations in neuronal function, for example related to ADHD and the use of common neurostimulants. These examples illustrate the important core mechanisms and provide learning opportunities, which might address different interests, skills and socially meaningful phenomena. At the same time, they allow students to choose learning contexts that are personally relevant to them, rather than following teachers' instructions or fulfilling mandatory curricular requirements. This might facilitate identification with the subject und in turn promote more self-determined motivation (such as profiles #3 and especially #4). From a broader educational policy perspective, curricular frameworks should therefore offer greater scope for taking students' interests and needs into account. Thirdly, biology education plays an indispensable role in the acquisition of well-founded scientific literacy and contributes to a responsible way of living (Kultusministerkonferenz, 2020). This becomes particularly evident in topics such as sex education, immunobiology, and genetics. These three examples illustrate some of the qualities inherent to the subject of biology, which should be utilized to support self-determined motivation.

However, whether and the extent to which this potential is exploited fundamentally depends on the learning environment (Niemiec and Ryan, 2009). In this context, the teacher plays a key role in promoting self-determined learning settings (Ahmadi et al., 2023; Reeve, 2002; Ryan and Deci, 2017). Various studies have shown the positive effects of an autonomy-supportive teaching style on different motivational and cognitive outcomes (Großmann and Wilde, 2020; Soenens and Vansteenkiste, 2005; Vansteenkiste et al., 2004). A recent study by Ahmadi et al. (2023) offered a framework comprising differentiated teaching strategies and specific instructions on how to foster students' basic psychological need satisfaction and avoid frustration thereof. They also provided detailed examples to illustrate these strategies. In biology classes, autonomy-supportive teaching strategies have in particular been applied and evaluated (Großmann and Wilde, 2020). However, these studies do primarily focus on the teaching behavior without considering students' motivational predisposition. Taking into account the results of our study, the perception of the respective teaching method might also vary in terms of students' motivational profiles. This highlights a relevant direction for further investigation. Therefore, further studies should continue to focus on teaching strategies (see e.g., Ahmadi et al., 2023) that foster self-determined biology learning considering different motivational profiles as learning predisposition.

## Data Availability

The raw data supporting the conclusions of this article will be made available by the authors, without undue reservation.
