# Supplementary material for: Motivational profiles regarding biology amongst German upper secondary students and associations with perceived basic need satisfaction and pressure
Source: Front Psychol. 2026 Mar 4;17:1588742. doi: 10.3389/fpsyg.2026.1588742 (PMC12996076; doi:10.3389/fpsyg.2026.1588742)
Supplement: Supplementary file 1 [file Table_1.DOCX]

**Tab. 1.** Descriptive summary for learner´s use of ODER in Case 1, across the counselling cycle and individual sessions, reporting counts, anchor type, primary function, sequential slot, prosodic design, discourse function of the sequence and next-turn continuation.

| **LEARNER CASE 1** | | | | | | | |
| --- | --- | --- | --- | --- | --- | --- | --- |
| **Sessions** | **1** | **2** | **3** | **4** | **5** | **6** | **7** |
|  | | | | | | | |
| **Turn-final ODER counts (90 occurrences)** | 14 | 15 | 21 | 8 | 14 | 15 | 3 |
| **Primary functional value** | | | | | | | |
| - confirmation prompt / request for verification  - pragmatic mitigation  - call for empathic alignment | 14 | 15 | 16 | 1 | 5 | 8 |  |
|  |  | | 2 | 3 | 4 | 3 | 2 |
|  |  |  | 3 | 4 | 5 | 4 | 1 |
| **Prosodic design** | | | | | | | |
| Phrasing: separated  Contour: rising  Upstep in relation to preceding utterance | 5 | 6 | 10 | 5 | 8 | 9 | 2 |
|  | 10 | 11 | 17 | 6 | 12 | 13 | 3 |
|  |  | 1 | 3 | 4 | 7 | 9 | 2 |
| **Preceding utterance** | | | | | | | |
| - question  **-** fragment  **-** statement | 14 | 11 | 6 |  | 1 |  |  |
|  |  | 2 | 9 | 1 | 1 |  |  |
|  |  | 2 | 6 | 7 | 12 | 15 | 3 |
| **Discourse function of the sequence** | | | | | | | |
| **-** Report on learning and planning  - Self-assessment  **-** General knowledge statements about learning  **-** Illustration (intentions, goals)  **-** Self-disclosures (analogies to prior experiences)  - Language-related reflections and hypothesis  - Checking understanding  - Narratives (small talk, language process, learning episodes)  - Self-initiated introduction of new, dissonant information  - Self-repair (on text)  - Alternative suggestions  - Disagreement | 1 | 1 |  |  |  | 1 |  |
|  |  |  | 3 | 4 | 5 | 4 | 2 |
|  | 3 | 3 | 3 |  |  | 2 |  |
|  | 1 | 1 | 1 |  |  |  |  |
|  | 1 | 1 |  |  |  |  |  |
|  | 2 | 3 | 4 | 1 | 4 | 3 |  |
|  | 2 | 2 | 2 |  |  |  |  |
|  | 2 | 3 | 4 | 1 | 1 | 2 |  |
|  | 1 |  | 2 |  | 1 | 1 | 1 |
|  | 1 | 1 | 2 |  | 1 |  |  |
|  |  |  |  | 2 | 1 | 1 |  |
|  |  |  |  | 1 | 1 | 1 |  |
| **Sequential slot parameters** | | | | | | | |
| - Temporary self-interruption  - Transitional / bridging (topic transition, activity boundary)  - Pre-closing relevance  - Preference organization: dispreferred move  - Post-candidate understanding  - Self-initiative continuation  - pre-expansion  - post-expansion | 5 | 8 | 10 | 1 | 1 | 1 |  |
|  | 1 |  | 1 | 2 | 2 | 4 |  |
|  | 1 |  | 1 |  | 2 | 1 |  |
|  | 1 |  | 2 | 3 | 4 | 3 | 2 |
|  | 2 | 4 |  |  | 2 | 1 |  |
|  | 2 | 2 | 2 |  | 2 | 3 | 2 |
|  |  |  |  |  |  |  |  |
|  | 2 | 1 |  | 2 | 1 | 2 | 1 |
| **Response - next turn continuation** | | | | | | | |
| - co-constructive repair  - confirmation (+positive assessment)  - a hedged confirmation  - agreement + comment  - expansion, elaboration  - epistemic uncertainty, relativization  - continuation  - a hedged rejection  - affiliative response | 1 | 4 | 3 |  |  | 2 |  |
|  | 8 | 8 | 12 | 1 | 5 | 5 | 2 |
|  | 1 | 1 | 1 |  | 2 | 1 |  |
|  | 1 |  |  | 2 | 1 |  |  |
|  | 1 | 1 | 2 | 2 | 3 | 3 |  |
|  | 1 | 1 |  | 1 |  | 1 |  |
|  |  |  | 2 |  |  |  |  |
|  | 1 |  |  |  |  |  |  |
|  |  |  | 3 | 2 | 3 | 3 | 1 |

**Tab. 2** Descriptive summary for learner´s use of ODER in Case 2, across the counselling cycle and individual sessions, reporting counts, anchor type, primary function, sequential slot, prosodic design, discourse function of the sequence and next-turn continuation.

| **LEARNER CASE 2** | | | | | | | |
| --- | --- | --- | --- | --- | --- | --- | --- |
| **Sessions** | **1** | **2** | **3** | **4** | **5** | **6** | **7** |
|  | | | | | | | |
| **Turn-final ODER counts (35 occurrences)** | 1 | 10 | 5 | 4 | 9 | 5 | 1 |
| **Primary functional value** | | | | | | | |
| - confirmation prompt / request for verification  - pragmatic mitigation | 1 | 10 | 5 | 2 | 7 | 4 | 1 |
|  |  | | | 2 | 2 | 1 |  |
| **Prosodic design** | | | | | | | |
| Phrasing: separated  Contour: rising  Upstep in relation to preceding utterance | 1 | 7 | 4 | 3 | 7 | 5 | 1 |
|  | 1 | 9 | 5 | 4 | 8 | 5 | 1 |
|  |  | | | 2 | 2 | 3 |  |
| **Preceding utterance** | | | | | | | |
| - question  **-** fragment  **-** statement | 1 | 8 | 2 | 1 | 2 |  |  |
|  |  | 1 | 1 | 1 | 1 | 1 |  |
|  |  | 1 | 2 | 3 | 5 | 4 | 1 |
| **Discourse function of the sequence** | | | | | | | |
| **-** General knowledge statements about learning  **-** Illustration (intentions, goals)  **-** Self-disclosures (analogies to prior experiences)  - Language-related reflections and hypothesis  - Checking understanding  - Narratives (small talk, language process, learning episodes)  - Self-initiated introduction of new, dissonant information  - Self-repair (on text) |  | 1 | 2 |  | 1 |  |  |
|  | 1 |  |  |  | 1 |  |  |
|  |  |  |  |  | 1 |  | 1 |
|  |  |  |  | 2 | 1 | 2 |  |
|  |  | 4 | 1 |  | 1 | 1 |  |
|  |  | 2 | 1 |  | 1 | 1 |  |
|  |  |  |  | 2 | 1 | 1 |  |
|  |  | 3 | 1 |  | 2 |  |  |
| **Sequential slot parameters** | | | | | | | |
| - Temporary self-interruption  - Transitional / bridging (topic transition, activity boundary)  - Pre-closing relevance  - Preference organization: dispreferred move  - Post-candidate understanding  - Self-initiative continuation  - pre-expansion  - post-expansion | 1 | 4 | 2 |  | 1 |  |  |
|  |  |  |  | 1 | 3 | 1 |  |
|  |  |  | 1 |  |  | 1 |  |
|  |  |  |  | 2 | 2 |  |  |
|  |  | 4 | 1 |  | 1 | 1 |  |
|  |  |  | 1 |  | 1 | 1 |  |
|  |  |  |  |  |  |  |  |
|  |  | 1 |  | 1 | 2 | 1 |  |
| **Response - next turn continuation** | | | | | | | |
| - co-constructive repair  - confirmation (+positive assessment)  - a hedged confirmation  - agreement + comment  - expansion, elaboration  - epistemic uncertainty, relativization  - continuation  - rejection | 1 | 4 | 1 | 1 | 1 | 1 | 1 |
|  |  | 2 | 2 | 1 | 2 | 2 |  |
|  |  | 2 | 1 | 1 | 1 |  |  |
|  |  |  |  |  | 1 | 1 |  |
|  |  |  | 1 | 1 | 4 | 1 |  |
|  |  | 1 |  |  |  |  |  |
|  |  | 1 |  |  |  |  |  |
|  |  |  |  |  |  |  |  |

**Tab. 3** Descriptive summary for advisor´s use of ODER in Case 1, across the counselling cycle and individual sessions, reporting counts, anchor type, primary function, sequential slot, prosodic design, discourse function of the sequence and next-turn continuation.

| **ADVISOR CASE 1** | | | | | | | |
| --- | --- | --- | --- | --- | --- | --- | --- |
| **Sessions** | **1** | **2** | **3** | **4** | **5** | **6** | **7** |
|  | | | | | | | |
| **Turn-final ODER counts (18 occurrences)** | - | 2 | 3 | 2 | 6 | 1 | 4 |
| **Primary functional value** | | | | | | | |
| - delegation of collaborative response (epistemic self-downgrading)  - pragmatic mitigation  - shaping narratives |  | 2 | 2 | 1 | 2 |  | 1 |
|  |  |  | 1 | 1 | 2 | 1 | 1 |
|  |  |  |  |  | 2 |  | 2 |
| **Prosodic design** | | | | | | | |
| Phrasing: separated  Contour: rising  Upstep in relation to preceding utterance |  | 2 | 2 | 1 | 4 | 1 | 3 |
|  |  | 2 | 2 | 2 | 5 | 1 | 3 |
|  |  | - | - | 1 | 3 | 1 | 2 |
| **Preceding utterance** | | | | | | | |
| - question  **-** fragment  **-** statement |  | 1 | 1 | 1 | 2 | 1 | 1 |
|  |  |  | 1 |  | 1 |  |  |
|  |  | 1 | 1 | 2 | 3 |  | 2 |
| **Discourse function of the sequence** | | | | | | | |
| **-** corrective text-based feedback  - shaping language reflections & hypothesis  - shaping and supporting narratives  - re-shaping reflection in action (after explicit advisory resistance)  - initiating / shaping reflection on the advisory process  - responsive facilitation of a practical exercise sequence  - second advisory attempt (after previous resistance) |  | 2 |  |  | 2 |  | 1 |
|  |  |  | 1 |  |  |  |  |
|  |  |  |  |  | 2 |  | 2 |
|  |  |  | 1 | 1 | 1 | 1 |  |
|  |  |  |  |  |  |  | 1 |
|  |  |  | 1 | 1 |  |  |  |
|  |  |  |  |  | 1 |  |  |
| **Sequential slot parameters** | | | | | | | |
| - sequence introduction  - pre-expansion  - post-expansion  - following up with concluding / pointing  - transitional / bridging (topic transition, activity boundary)  - Pre-closing relevance  - Preference organization: dispreferred move  - Post-candidate understanding  - Resistance in pre-field |  | 1 |  |  | 1 |  | 1 |
|  |  |  |  |  |  |  |  |
|  |  | 1 |  |  | 2 |  | 1 |
|  |  |  | 1 |  | 2 |  |  |
|  |  |  | 1 | 1 | 1 | 1 | 1 |
|  |  |  | 1 | 1 |  |  | 1 |
|  |  |  |  |  |  |  |  |
|  |  |  | 1 | 1 | 2 | 1 |  |
| **Response - next turn continuation** | | | | | | | |
| - co-constructive repair  - confirmation (+positive assessment)  - a hedged confirmation  - agreement + comment  - expansion, elaboration  - epistemic uncertainty, relativization  - continuation  - hedged rejection and elaboration |  | 1 |  | 1 | 1 |  |  |
|  |  |  | 1 |  | 1 |  | 3 |
|  |  | 1 |  |  | 1 |  | 1 |
|  |  |  |  |  |  |  |  |
|  |  |  | 1 | 1 | 1 |  |  |
|  |  |  |  |  |  |  |  |
|  |  |  |  |  | 1 |  |  |
|  |  |  | 1 |  | 1 | 1 |  |

**Tab. 4** Descriptive summary for advisor´s use of ODER in Case 2, across the counselling cycle and individual sessions, reporting counts, anchor type, primary function, sequential slot, prosodic design, discourse function of the sequence and next-turn continuation.

| **ADVISOR CASE 2** | | | | | | | |
| --- | --- | --- | --- | --- | --- | --- | --- |
| **Sessions** | **1** | **2** | **3** | **4** | **5** | **6** | **7** |
|  | | | | | | | |
| **Turn-final ODER counts (42 occurrences)** | 2 | 3 | 6 | 5 | 4 | 14 | 8 |
| **Primary functional value** | | | | | | | |
| - delegation of collaborative response (epistemic self-downgrading)  - pragmatic mitigation  - shaping narratives  - mitigating empathic projections  - in relation to practices of *bringing home the point* |  | 1 | 1 | 1 |  | 4 | 1 |
|  |  |  |  |  | 1 | 3 |  |
|  | 1 | 1 | 2 | 2 | 1 | 2 | 2 |
|  |  |  | 2 | 1 | 1 | 2 | 3 |
|  | 1 | 1 | 1 | 1 | 1 | 3 | 2 |
| **Prosodic design** | | | | | | | |
| Phrasing: separated  Contour: rising  Upstep in relation to preceding utterance | 2 | 2 | 4 | 5 | 4 | 13 | 7 |
|  | 2 | 3 | 5 | 5 | 4 | 13 | 8 |
|  |  | 1 | 3 | 3 | 3 | 8 | 5 |
| **Preceding utterance** | | | | | | | |
| - question  **-** fragment  **-** statement | 2 | 3 | 2 | 2 | 2 | 2 | 2 |
|  |  |  | 1 | 1 |  | 2 |  |
|  |  |  | 3 | 2 | 2 | 10 | 6 |
| **Discourse function of the sequence** | | | | | | | |
| - corrective text-based feedback  - shaping language learning reflections & hypothesis  - shaping and supporting narratives  - uptake of a prior conversational element to initiate elaboration  **-** responsive facilitation of a practical exercise sequence  - language contrastive reflections |  | 1 |  |  |  |  |  |
|  | 1 | 1 | 3 | 3 | 3 | 3 | 3 |
|  | 1 | 1 | 2 | 2 | 1 | 4 | 4 |
|  |  |  | 1 |  |  |  | 1 |
|  |  |  |  |  |  | 4 |  |
|  |  |  |  |  |  | 3 |  |
| **Sequential slot parameters** | | | | | | | |
| - sequence introduction  - pre-expansion  - post-expansion  - following up with concluding / pointing  - following up with inferential continuer  - transitional / bridging (topic transition, activity boundary)  - pre-closing relevance |  |  | 1 |  |  | 1 |  |
|  |  |  |  |  |  |  |  |
|  |  |  |  | 1 |  |  | 2 |
|  |  | 2 | 2 | 1 | 2 | 4 | 3 |
|  | 2 | 1 | 2 | 1 | 2 | 5 | 3 |
|  |  |  | 2 | 2 |  | 2 |  |
|  |  |  | 1 |  |  | 2 |  |
| **Response - next turn continuation** | | | | | | | |
| - co-constructive repair  - confirmation (+positive assessment)  - a hedged confirmation  - agreement + comment  - expansion, elaboration  - epistemic uncertainty, relativization  - continuation  - hedged rejection and elaboration |  |  |  |  |  | 1 |  |
|  |  |  |  | 1 |  | 2 | 1 |
|  | 2 | 1 | 1 |  |  | 1 | 1 |
|  |  |  |  |  |  |  |  |
|  |  | 2 | 4 | 5 | 4 | 8 | 6 |
|  |  |  |  |  |  |  |  |
|  |  |  |  |  |  | 1 |  |
|  |  |  | 1 |  |  | 1 |  |
